# A Dual-Butterfly Structure Gyroscope

**DOI:** 10.3390/s17122870

**Published:** 2017-12-11

**Authors:** Xiangming Xu, Dingbang Xiao, Wenyin Li, Qiang Xu, Zhanqiang Hou, Xuezhong Wu

**Affiliations:** College of Mechatronics Engineering and Automation, National University of Defense Technology, Changsha 410073, China; xuxiangming15@nudt.edu.cn (X.X.); liwenyin12365@163.com (W.L.); xuqiang09@nudt.edu.cn (Q.X.); houzhanqiang@nudt.edu.cn (Z.H.); xzwu@nudt.edu.cn (X.W.)

**Keywords:** quad differential, butterfly gyroscope, coupling

## Abstract

This paper reports a dual-butterfly structure gyroscope based on the traditional butterfly structure. This novel structure is composed of two butterfly structures, each of which contains a main vibrational beam, four proof masses, and a coupling mechanism. The coupling mechanism in this proposed structure couples the two single butterfly structures and keeps the driving mode phases of the two single butterfly gyroscopes exactly opposite, increasing the double difference of traditional butterfly gyroscopes to a quad difference, which has the potential advantage of improving bias instability and g-sensitivity. The gyroscope was fabricated using a standard microfabrication method and tested in laboratory conditions. The experimental results show a Q-factor of 10,967 in driving mode and there were two peaks in the frequency responses curve of sensing direction due to unavoidable fabrication errors. Scale factor and bias instability were also measured, reaching a scale factor of 10.9 mV/°/s and a bias instability of 10.7°/h, according to the Allan Variance curve.

## 1. Introduction

Micro-gyroscopes are important Micro-electromechanical System (MEMS) inertial sensors with broad applications in civil and military fields [1]. The significance of gyroscopes has attracted persistent worldwide efforts to this research area and different kinds of micro-gyroscopes have been developed and researched deeply, including tuning fork gyroscopes [2,3], disk resonator gyroscopes (DRG) [4,5], and hemispherical resonator gyroscopes (HRG) [6,7]. Among them, butterfly gyroscopes have played an important role ever since they were developed in 1999 by IMEGO institute [8]. The conventional butterfly gyroscope, with four proof masses and a main vibrational beam, enables masses to vibrate along the planar direction using the normal electrostatic force from the bottom electrodes. The detection of angular rate was achieved through the detection of capacitance variation between the silicon structure and the bottom electrodes. As the two masses vibrate in anti-phase, the offset is smaller and the gyroscope is less sensitive to linear and angular external vibrations. Since then, researchers have done a lot of work based on this structure due to its brilliant advantages. A bulk micro-machined angular rate sensor based on the butterfly-gyro structure was developed by Sensonor Technologies in 2001 [9]. A series of works were also carried out by our research group [10,11,12]. They demonstrated advantages in batch-fabrication costs and high performance due to the double differential structure and simple fabrication process.

Structure optimization is an effective way to improve the performance of MEMS devices, and it has been widely used in MEMS device design. Anti-phase operation of tuning-fork structures is a common method for improving the performance of gyroscopes in terms of bias instability and g-sensitivity [13]. To achieve anti-phase operation, an anchored ring spring was first used in a SOI-MEMS tuning fork gyroscope developed by K. Azgin from the Middle East Technical University [14]. The proposed gyroscope operates in a fully anti-phase driving mode and achieves a bias instability of 200°/h. A novel level coupling mechanism was also developed and used in a series of tuning fork gyroscopes developed by UC Irvine to achieve anti-phase oscillation in both driving and sensing modes [15,16,17,18], and this kind of gyroscope was reported to reach an extremely high level of performance with a bias instability of 0.09°/h [19]. Some simulations have also been done by Guan to verify the vibration sensitivity reduction of a tuning fork gyroscope when using two types of coupling mechanisms to reach anti-phase vibration [20,21]. The traditional butterfly gyroscope, as mentioned above, also works in anti-phase mode in both driving direction and sensing direction in terms of the four masses. However, from the point of the vibrational beam, its vibration is asymmetric and not in anti-phase.

In this paper, some improvements to the structure of traditional butterfly gyroscopes have also been made to realize fully symmetrical and anti-phasic operation, so as to further increase the performance of butterfly gyroscopes. Two identical butterfly gyroscopes were coupled through a novel coupling mechanism which enables the two parts vibrate synchronously in opposite phases. The coupling mechanism also makes the in-phase frequency much higher than the working anti-phase frequency, which enables the gyroscope to work with less influence from low frequency environmental noise. Besides, through more times of difference (from two to four), disturbances and shock from the external environment will be offset by the opposite movement. Therefore, performance in terms of bias instability will potentially increase. Meanwhile, sensitivity will increase because of the doubled detection capacitance.

## 2. Design and Working Principle

### 2.1. Structure Design

**Figure 1:**
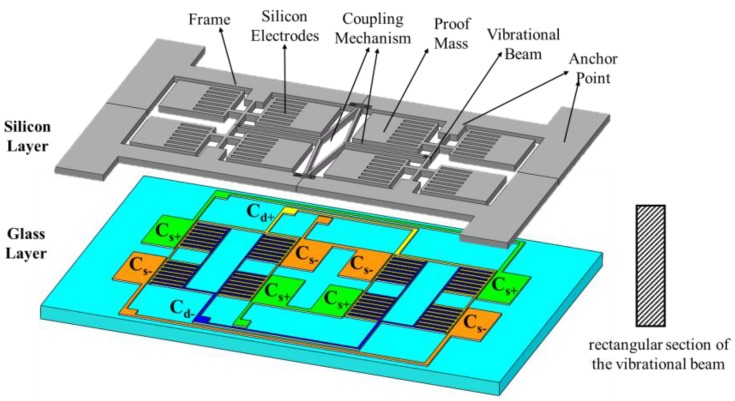
Schematic of the designed gyroscope.

The proposed quad differential butterfly gyroscope consists of two layers: a main sensitive silicon structure and a glass substrate which is bonded onto a silicon layer through the anchor point. All the metal electrodes were fabricated on the glass layer, and there is a gap between the metal electrodes in the glass and silicon structures. As depicted in Figure 1, the silicon structure is made from a 240 µm thick crystalline silicon wafer and consists of eight proof masses, two main vibrational beams, a frame, silicon electrodes with a comb structure, and a coupling mechanism. The coupling mechanism serves its aim to connect two single butterfly gyroscopes together and achieves mode ordering that separates the in- and anti-phase driving mode frequencies.

In the 500 µm thick glass layer, there are eight sensing electrode plates just beneath the eight proof masses, which are used to detect the sense mode oscillation of the proof masses. The sensing electrodes are shown in Figure 1 as $C_{s+}$ and $C_{s-}$. They are connected respectively to four pads to make sure the total capacitance variance $\Delta C_{s}=C_{s+}-C_{s-}$ and they are sensitive to the anti-phase rotation in sensing mode. There are also several comb-driving electrodes just aligned with the silicon comb electrodes in the silicon structure, which are used to excite the driving mode oscillation. As shown in Figure 1, the driving electrodes are connected to two pads shown as $C_{d+}$ and $C_{d-}$, and voltages are applied on the pads to drive the gyroscope.

**Figure 2:**
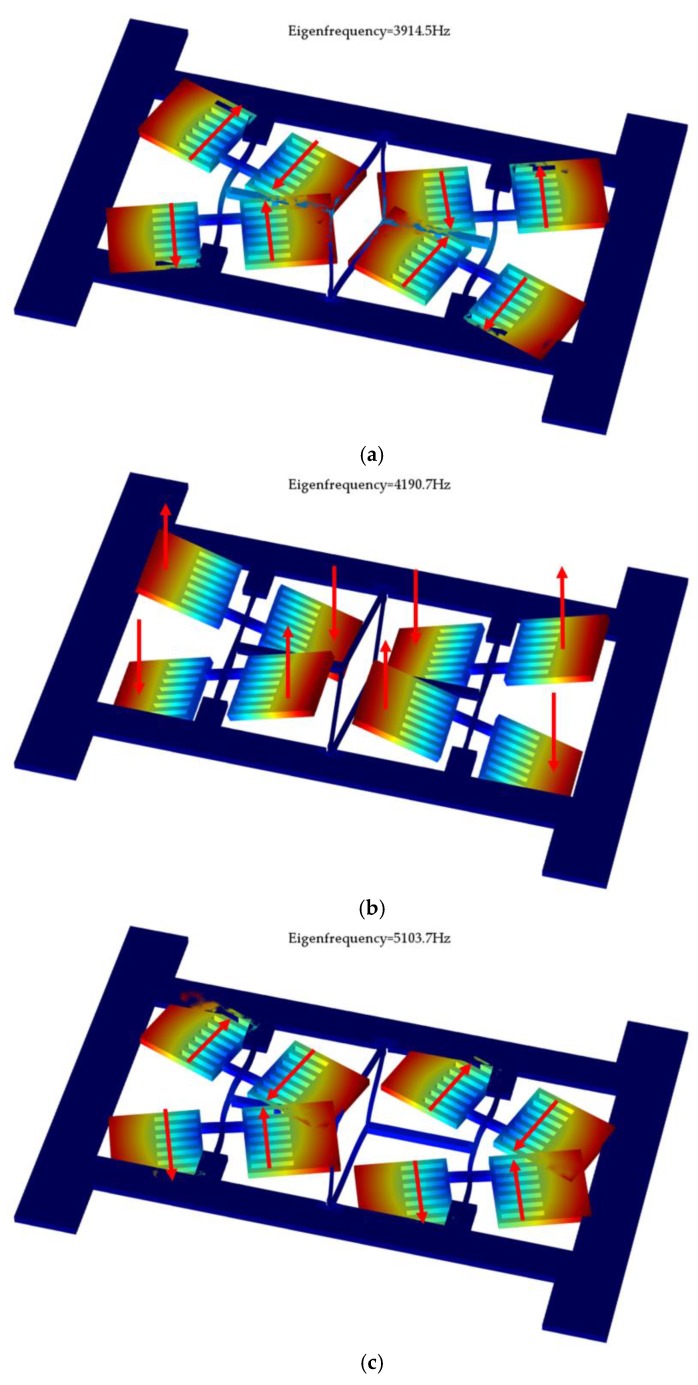
Modal analysis of the proposed silicon structure: (**a**) driving mode, (**b**) sensing mode, and (**c**) disturbing mode.

The modal analysis of this proposed gyroscope was carried out using COMSOL Multiphysics 5.2, and the simulation results are shown in the Figure 2, including the driving mode, sensing mode, and disturbing mode.

The driving mode is the anti-phase motion of two butterfly gyroscopes, each of which operates in the anti-phase swing oscillation of the masses in the gyroscopic plane caused by the bending of the vibrational beam. The sensing mode is also the anti-phase motion of two butterfly gyroscopes, each of which operates in the anti-phase oscillation of the masses out of the gyroscopic plane caused by torsion of the vibrational beam when Coriolis force is produced. Through detecting the variation in capacitance between the bottom electrodes in the glass layer and the proof masses, the input angular rate can be measured. The disturbing mode, which is also called the in-phase driving mode, is the in-phase motion of two butterfly gyroscopes.

As has been mentioned above, the in-phase driving mode has to be avoided to decrease the effects of external vibration and disturbance, thus a large frequency separation between the in- and anti-phase modes is needed [22,23]. However, a high driving mode frequency will reversely result in a low scale factor of gyroscope. Therefore, an improved mode ordering that makes the frequency of in-phase driving much higher than anti-phase driving is necessary and will potentially enhance the performance of the gyroscope. The proposed design shows that the frequency of these three modes are driving mode with 3914.5 Hz, sensing mode with 4190.7 Hz, and disturbing mode with 5103.7 Hz. It can be seen from this results that the coupling mechanism successfully separates the two driving modes.

### 2.2. The Principle of Tangential-Drive

Using the dry-etching processing method, the vibration structure can obtain a rectangular section vibration beam rather than a traditional one, which can change its driving method from a vertical force to a horizontal force. Due to the rectangular section vibration beam, the driving method can be changed from a vertical driving force to a tangential driving force. Through a tangential driving force, the driving amplitude will no longer be limited by the initial capacitive gap so that we can further increase the vibration amplitude to enhance the sensitivity of gyroscope. Also, there will be no obvious vertical displacement in the sense direction when the vibration structure is driven in the drive mode, so the initial capacitive gap can be further reduced, so as to increase the sensitivity of the gyroscope.

**Figure 3:**
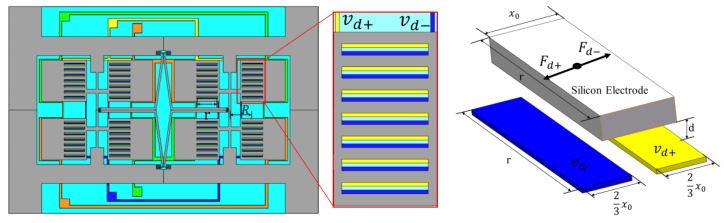
Schematic of the electrode alignment and driving force.

In order to achieve a tangential driving force, a type of electrode with a comb structure was designed on the mass of the silicon structure and the corresponding electrodes were designed on the glass layer to generate a tangential driving force. When the vibration structure is not totally aligned with glass plate, it will produce tangential electrostatic forces, as shown in Figure 3.

The drive voltages $V_{d+}$ and $V_{d-}$ are applied on the two pads of the driving electrodes, and the silicon structure is connected to ground. $V_{d+}$ and $V_{d-}$ are

(1) $$\begin{array}{l} V_{d+}=V_{dc}+V_{ac}\sin\omega_{d}t\\ V_{d-}=V_{dc}\;-\;V_{ac}\sin\omega_{d}t \end{array},$$

where $V_{dc}$ is the DC bias voltage, $V_{ac}$ is the magnitude of the AC component of the driving voltage, and $\omega_{d}$ is the frequency of the driving mode of the gyroscope.

Then, the produced tangential electrostatic forces by $V_{d+}$ and $V_{d-}$ are [24]:

(2) $$F_{d+}=\frac{r\varepsilon_{0}}{2d}{\left(V_{dc}+V_{ac}\sin\omega_{d}t\right)}^{2}\;F_{d-}=\frac{r\varepsilon_{0}}{2d}{\left(V_{dc}-V_{ac}\sin\omega_{d}t\right)}^{2}$$

where r is the length of the comb, d is the gap distance between the two layers, and $\varepsilon_{0}$ is the dielectric constant.

Thus, the total resultant tangential driving force and moment acting on a proof mass are:

(3) $$F_{d}=N\times (F_{d+}-F_{d-})=\frac{2Nr\varepsilon_{0}V{}_{dc}V_{ac}\sin\omega_{d}t}{d}$$

(4) $$M_{dz}=F_{d}R=\frac{2NRr\varepsilon_{0}V{}_{dc}V_{ac}\sin\omega_{d}t}{d}$$

where N is the amount of the comb and R is the distance between the electrode and the rotating center of the driving mode.

$M_{dz}$ bends the vibrational beam so the drive mode oscillation is generated, and the proof mass will vibrate within the plane but not out of the plane. In this way, the gap between the two layers can be reduced to increase the driving force and enhance the sensitivity of the gyroscope [25].

### 2.3. Analysis of the Coupling Mechanism

**Figure 4:**
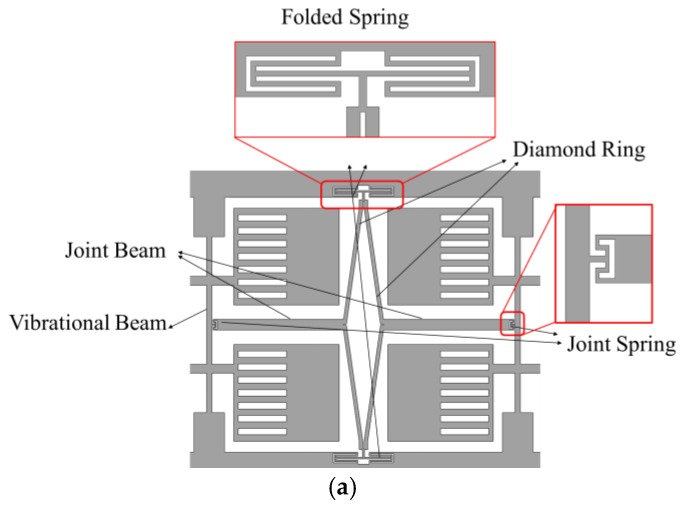

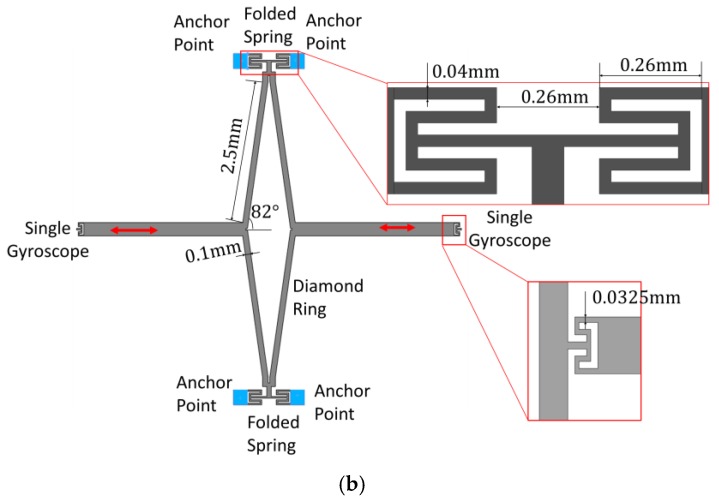
(**a**) Schematic diagram of the coupling mechanism (**b**) The main size of the coupling mechanism.

As mentioned before, an improved mode ordering that shifts the frequency of in-phase driving much higher than the anti-phase driving frequency is necessary. In this proposed structure, it is achieved by novel coupling mechanism, as depicted in Figure 4a, which is composed of a diamond ring, two folded springs embedded in the frame, two joint beams, and two joint springs connecting to the vibrational beams. Figure 4b shows the main size of the adopted mechanism in this research.

The coupling mechanism aims to make the two single gyroscopes vibrate synchronously in anti-phase to enhance detection sensitivity and also reduce sensitivity to other disturbances. In this design, a diamond ring and two folding springs are utilized. The diamond ring makes sure that the transversal displacement of the two joint beams has a direct and large influence on the deformation of the folded spring compared to the two parallel beams, so that the stiffness of the whole gyroscope can be easily adjusted by the folded springs in different conditions. When the two parts vibrate in anti-phase, the springs are squeezed or stretched in the direction orthogonal to that movement. This is the lowest frequency mode of the spring, therefore the frequency of the anti-phase mode is lower than that of the in-phase mode [14]. In this way, low frequency noise in the environment will nearly not result in in-phase disturbing movements, thus increasing performance in terms of instability bias.

**Figure 5:**
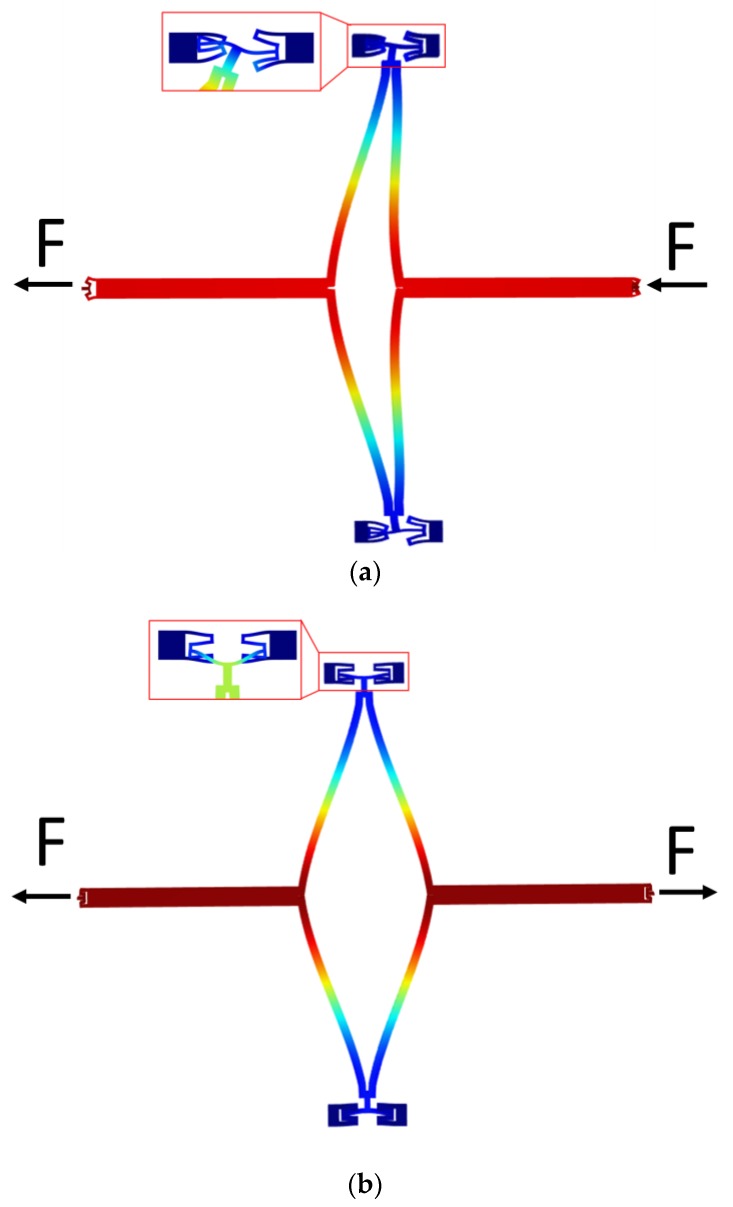
Simulation of stiffness of the coupling mechanism: (**a**) in-phase driving, (**b**) anti-phase driving.

The frequency separation is realized through different stiffnesses of the coupling mechanism in in- and anti-phase driving motion, therefore it is necessary to analyze the stiffness of this mechanism in different conditions. Because of the complexity of the theoretical arithmetic of this complicated structure, finite element method (FEM) simulation by COMSOL Multiphysics is utilized to simulate structure deformation when the mechanism works in different situations. As is shown in Figure 5, two equal forces are applied to the end of the joint springs to simulate the real conditions in driving mode. The two forces are in opposite directions in anti-phase driving and in the same direction in in-phase driving. Supposing the displacement of the end is x, the stiffness of this mechanism can then be calculated as:

(5) $$k=\frac{F}{x},$$

**Figure 6:**
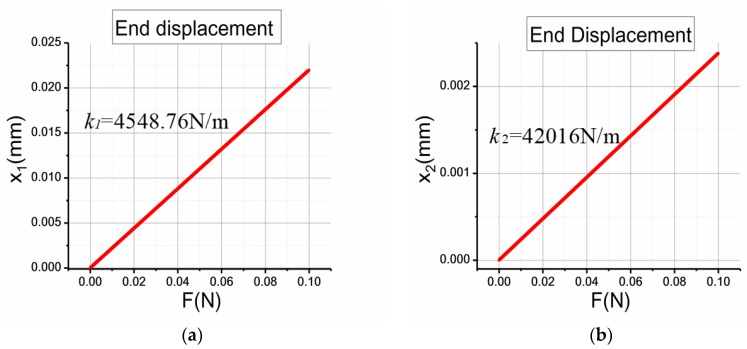
The stiffness of the coupling mechanism in the (**a**) anti-phase and (**b**) in-phase modes.

By applying different forces and calculating different displacements in COMSOL, the relationship between applied force and generated displacement is obtained and the stiffnesses of two conditions is depicted in Figure 6. It can be seen that the displacement of the end shows a linear relationship with the applied force. More importantly, the stiffness of the mechanism working in in-phase driving mode is much higher than that in anti-phase driving mode (about 10 times higher), which demonstrates that the proposed mechanism can achieve the goal of enlarging the stiffness of the in-phase driving mode and lowering the stiffness of the anti-phase mode.

### 2.4. Theoretical Analysis

**Figure 7:**
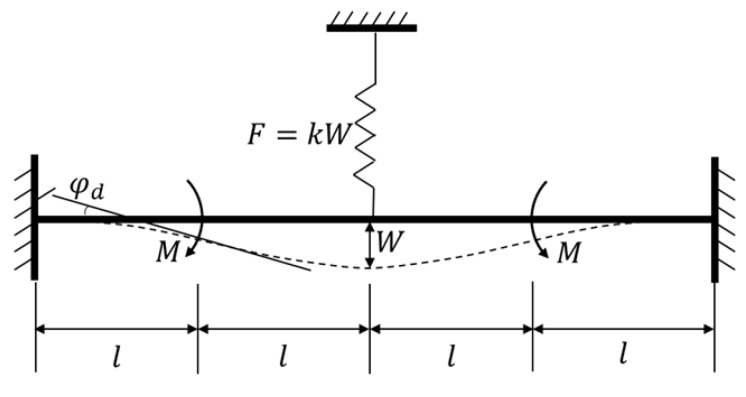
Theoretical model of the vibrational beam.

The silicon structure is fully symmetrical and it has been verified that the mechanism can be treated as a spring in terms of the liner relationship between applied force and generated displacement. To simplify the analysis, the main vibrational beam can be regarded as a clamped-clamped beam with a length of 4l, as depicted in Figure 7. The coupling mechanism is simplified to a spring with a stiffness of k that acts at the midpoint of the beam, and the generated elastic force can be defined as

(6) F=kW,

where W is the deflection of the beam at the midpoint, M is the produced moment by the tangential driving force of two proof masses, which acts on the 1/4 length-point of the beam, and the caused bending angle of this position is $\varphi_{d}$.

Supposing that the elastic force F acts on the beam alone, from knowledge on mechanics of materials [26], the generated deflection and bending angle can be calculated as:

(7) $$W_{1}=-\frac{Fl^{3}}{3EI}$$

(8) $$\varphi_{d1}=-\frac{Fl^{2}}{4EI},$$

where E is Young’s modulus of silicon and I is the principal moment of inertia of the vibrational beam, which can be written as:

(9) $$I=\frac{wh^{3}}{12},$$

Then supposing that the driving moment M acts alone, the generated deflection and bending angle can thus be calculated as:

(10) $$W_{2}=\frac{Ml^{2}}{2EI},$$

(11) $$\varphi_{d2}=\frac{Ml}{2EI},$$

Based on the superposition principle in the mechanics of materials [26], the actual deflection and bending angle are:

(12) $$W=W_{1}+W_{2}=-\frac{Fl^{3}}{3EI}+\frac{Ml^{2}}{2EI},$$

(13) $$\varphi_{d}=\varphi_{d1}+\varphi_{d2}=-\frac{Fl^{2}}{4EI}+\frac{Ml}{2EI}$$

Substituting Equation (6) into Equations (12) and (13), one obtains:

(14) $$F=\frac{3Mkl^{2}}{6EI+2kl^{3}},$$

(15) $$\varphi_{d}=\frac{Ml}{2EI}-\frac{3Mkl^{4}}{24E^{2}I^{2}+8EIkl^{3}}$$

Therefore, the bending stiffness of the vibrational beam can be calculated as:

(16) $$k_{d}=\frac{M}{\varphi_{d}}=\frac{M}{\frac{Ml}{2EI}-\frac{3Mkl^{4}}{24E^{2}I^{2}+8EIkl^{3}}}=\frac{2EI}{l}\left(1+\frac{3kl^{3}}{12EI+kl^{3}}\right),$$

The theoretical value of the driving mode frequency is written as:

(17) $$\omega_{d}=\sqrt{\frac{k_{d}}{I_{d}}}=\sqrt{\frac{2EI}{I_{d}l}\left(1+\frac{3kl^{3}}{12EI+kl^{3}}\right)},$$

where $I_{d}$ is the inertial moment of two proof masses in driving mode.

It has been simulated in 2.2 that the stiffness of the coupling mechanism in driving mode is:

Anti-phase driving mode: $k_{1}\;=\;4548.76\;N/m$
In-phase driving mode: $k_{2}\;=\;42,016\;N/m$

Therefore, the frequency ratio of anti-phase mode to in-phase mode can be calculated based on Equation (17):

(18) $$\frac{\omega_{d\_in}}{\omega_{d\_out}}\approx 1.339,$$

Therefore, the stiffness of the coupling mechanism has a direct effect on the frequency of the driving mode in this gyroscope, and it can also be deducted that the theoretical analysis result is in a good agreement with the simulation result in Section 2.1 in terms of the frequency ratio in two driving modes.

## 3. Fabrication Process

**Figure 8:**
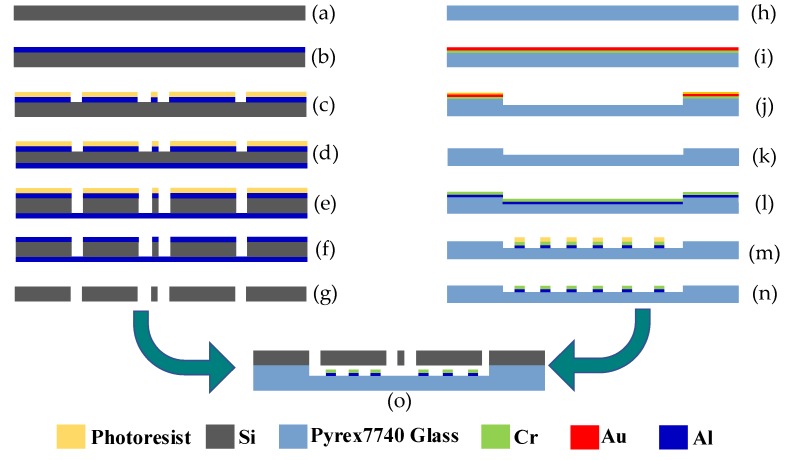
Fabrication process of the proposed gyroscope.

The proposed gyroscope was fabricated using a standard MEMS microfabrication process. As is shown in Figure 8, the fabrication process is mainly composed of three parts: the fabrication of the silicon structure, the fabrication of the electrodes in the glass substrate, and the bonding process of the two layers. In order to obtain a vibrational beam with a rectangular section, Deep Reactive Ion Etching (DRIE) was utilized as the etching method to form the silicon structure.

A detailed description is as follows: (a) the starting material, a 240 µm thick silicon wafer; (b) deposition of a layer of Al with a 100 nm thickness; (c) first UV photolithographing and etching of Al; (d) deposition of a layer of Al with a 100 nm thickness on another side of the silicon wafer; (e) DRIE etching to another side; (f) removal of photoresist; (g) etching and removal of Al layer on both sides; (h) the starting material, 500 µm thick Corning Pyrex 7740 glass; (i) depositing two layers of Au and Cr on the glass; (j) photolithographing and etching of Au and Cr, and then etching the glass to 5 µm using the remaining Au and Cr as a mask; (k) etching and removal of the remaining Au and Cr; (l) depositing two layers of Cr and Al; (m) photolithographing and etching Al and Cr to make the electrodes; (n) removal of the photoresist; (o) connecting the silicon structure with the glass substrate by anodic bonding.

In the silicon fabrication process, metal Al was used as the etching mask for DRIE for its extremely high selection ratio in DRIE, and it was also used in the back side of the silicon substrate during DRIE to avoid the universal “notching effect” [27]. In the process on the glass substrate, a Cr and Au layer was used as the mask for glass etching in buffered oxide etch (BOE) solution due to their high resistance when etched. When bonding the two layers together using anodic bonding, the high voltage applied to the two sides may lead to electrostatic adhesion failure. To avoid this failure, a layer of Cr was deposited on the front of the Al because it will not form a chemical bond in anodic bonding.

**Figure 9:**
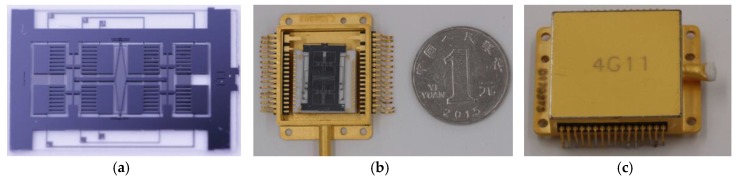
The fabricated MEMS gyroscope: (**a**) single gyroscope chip, (**b**) gyroscope chip wire-bonded to package, and (**c**) The vacuum-sealed gyroscope.

As was shown in Figure 9a, the bonded structure was then scribed using a tense-energy laser. The single gyroscope has dimensions of 10 mm × 18 mm. There are two diving electrode pads and four sensing electrode pads in the glass, and the silicon layer was connected to the ground. Single chips were then wire-bonded to package (Figure 9b). Finally, the package was vacuum-sealed under a pressure of $10^{-6}\mathrm{Pa}$ (Figure 9c).

## 4. Characterization

### 4.1. Frequency Response Testing in Driving and Sensing Direction

**Figure 10:**
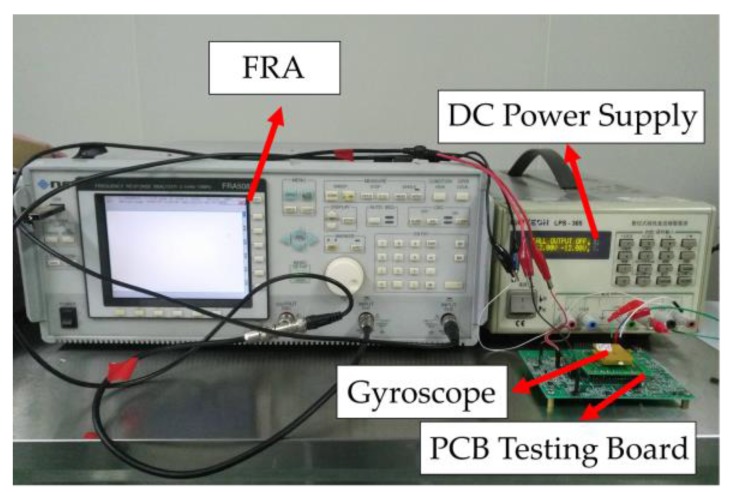
Frequency response testing setup.

**Figure 11:**
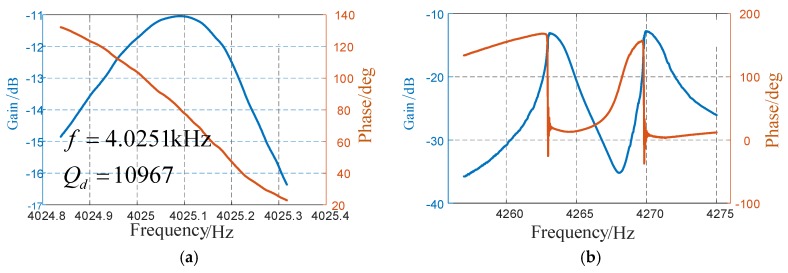
(**a**) Frequency response in driving direction (**b**) Frequency response in sensing direction.

The micro-gyroscope was firstly tested using a frequency response analyzer (NF FRA 5087, NF Corporation, Yokohama, Japan), DC-regulated power, and a model testing PCB board. The setup is shown in Figure 10. The test results are shown in Figure 11. In driving direction, the resonance frequency in anti-phase was 4025.1 Hz, and it can reach a Q-factor of 10,967 in this mode.

In sensing direction, it can be noted that there are two peaks around 4265 Hz, which is quite different from that in traditional butterfly gyroscopes. It is because there are always some fabrication errors during the manufacturing process, although the two parts are designed to be ideally identical. Also, the sensing mode of the two gyroscopes was not coupled, so they vibrate without influence from another. Therefore, the gain in sensing direction will reach its peak in resonance of both of the two parts. However, the gyroscope works at the anti-phase driving mode frequency, which is lower than, but not between, these two peaks. Therefore, when an external angular rate is applied to the gyroscope, the movement in sensing direction is still in anti-phase.

### 4.2. Measurement of Scale Factor and Bias Instability

**Figure 12:**
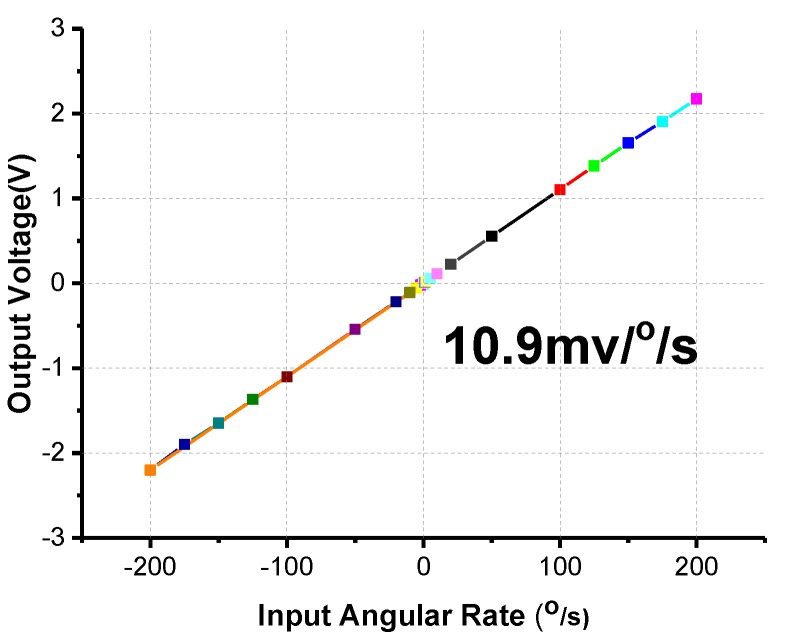
Scale factor testing of the gyroscope.

The scale factor is an important specification representing the ratio of a change in output to a change in the input angular rate. Under ambient temperature conditions, the fabricated gyroscope prototype was fixed on a precise rotating platform. A stable output was obtained over a range of angular rates, as shown in Figure 12. The scale factor of the fabricated gyroscope reached approximately 10.9 mV/°/s, and showed a measurement range of ±200°/s, and the scale factor nonlinearity is 1221 ppm.

**Figure 13:**
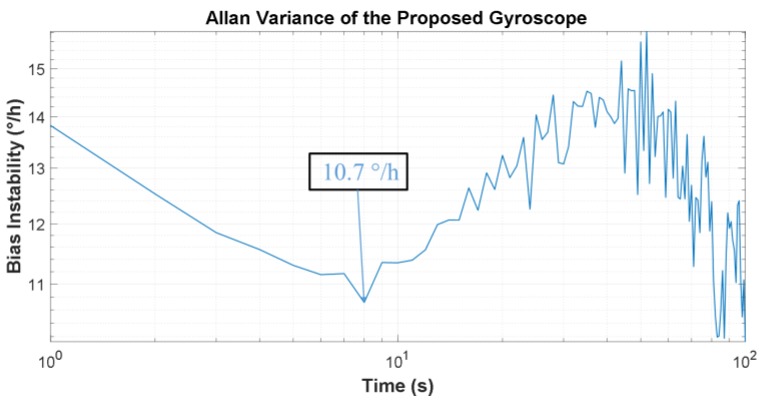
Allan variance curves of the proposed gyroscope.

Bias instability is another critical specification in gyroscope performance, referring to the output of the gyro at zero-angle input. A 1 h static test was performed at room temperature. The Allan variance was used to characterize the bias instability. The standard definition of bias instability used by inertial sensor manufacturers is the minimum point on the Allan variance curve (Figure 13). It can be calculated from the results that the bias instability is 10.7°/h.

### 4.3. The Quadrature Error

Quadrature error is induced by fabrication imperfections and will significantly influence the performance of a gyroscope. Even though there are several specific causes, quadrature error is mainly caused by the stiffness coupling from the driving mode to sensing mode, which should not happen in an ideal gyroscope [28]. In the proposed gyroscope, the measured quadrature error is 280°/s.

## 5. Conclusions

This paper reports the design, simulation, theoretical analysis, fabrication, and measurement of a quad differential butterfly gyroscope. The proposed gyroscope features a coupling mechanism which couples two single butterfly gyroscopes, and this structural design achieves the goal of increasing the in-phase mode frequency to be much larger than the anti-phase one by more than 1000 Hz. A lot of simulations and theoretical works were also done to get a full understanding of this novel structure and the theoretical results agree with the simulation results. The testing results of the fabricated gyroscope show that the design has a promising future. The scale factor is 10.9 mV/°/s with a measurement range of ±200°/s, and the scale factor nonlinearity is 1221 ppm. It also demonstrates a bias instability of 10.7°/h in terms of Allan Variance and a quadrature error of 280°/s.

More optimizations and measurements on the gyroscope are being done to achieve the expected goals of enhancing the stability and sensitivity to a higher level. Some new designs according to the wet-etching process are being done and we will try to achieve higher performance using closed-loop control. Also, the quadrature error suppression scheme is being developed using additional electrodes.
